# Supplementary material for: Uncovering Pathways Highly Correlated to NUE through a Combined Metabolomics and Transcriptomics Approach in Eggplant
Source: Plants (Basel). 2022 Mar 4;11(5):700. doi: 10.3390/plants11050700 (PMC8912549; doi:10.3390/plants11050700)
Supplement: Supplementary file 1 [file plants-11-00700-s001.zip › plants-1602087 R3 supplementary.pdf]

Figure S1. Interactive visualization system and detailed results from the pathway analysis in shoot are presented graphically for AM222, 67-3 and 305E40 vs. AM22: A) and B) to T0; C), D) and E) to T1; F), G), and H) to T2. Paths with FDR <0.05 and with a higher Impact value were highlighted. A P-value of less than 0.05, 0.01, and 0.001 was designated with one (*), two (**) or three (***) asterisks, respectively.

**A. Shoot_T0_67-3_*vs*_AM22; Alanine, aspartate and glutamate metabolism FDR = 0.047029 Impact = 0.64748**

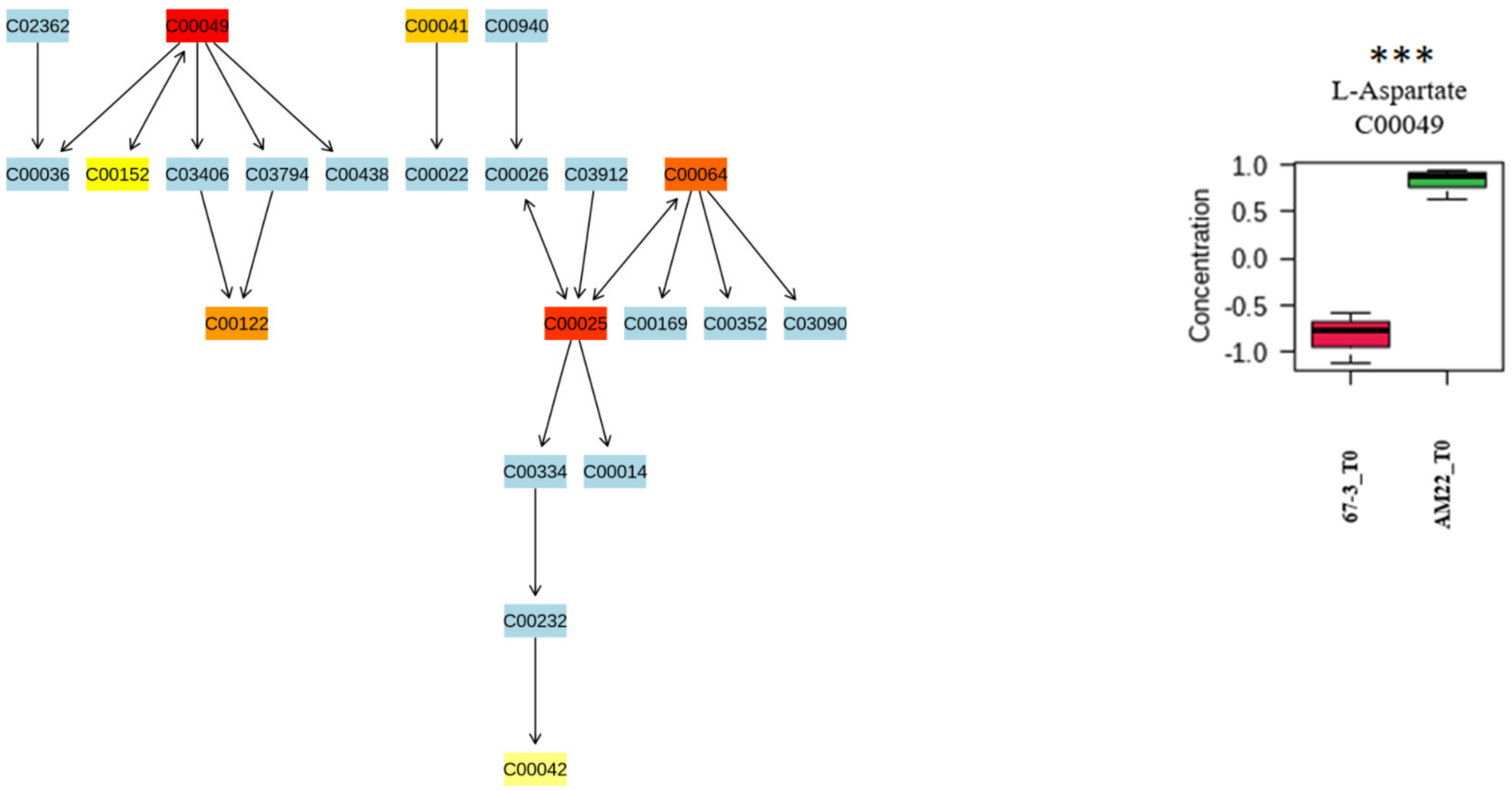

B. Shoot_T0_305E40_*vs*_AM22; Alanine, aspartate and glutamate metabolism FDR = 0.0094 Impact = 0.64748

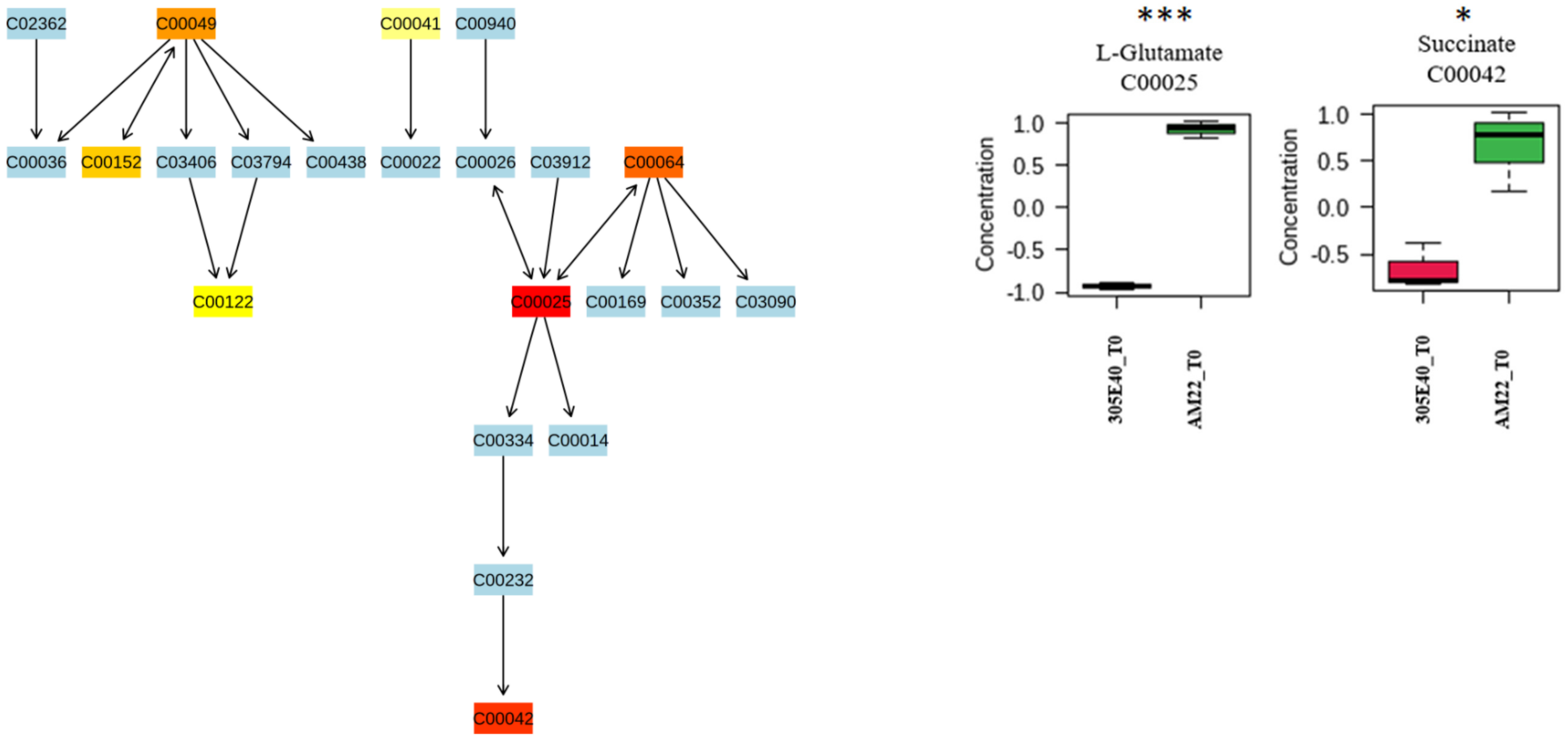

C. Shoot_T1_AM222_*vs*_AM22; Alanine, aspartate and glutamate metabolism FDR = 0.010616 Impact = 0.64748

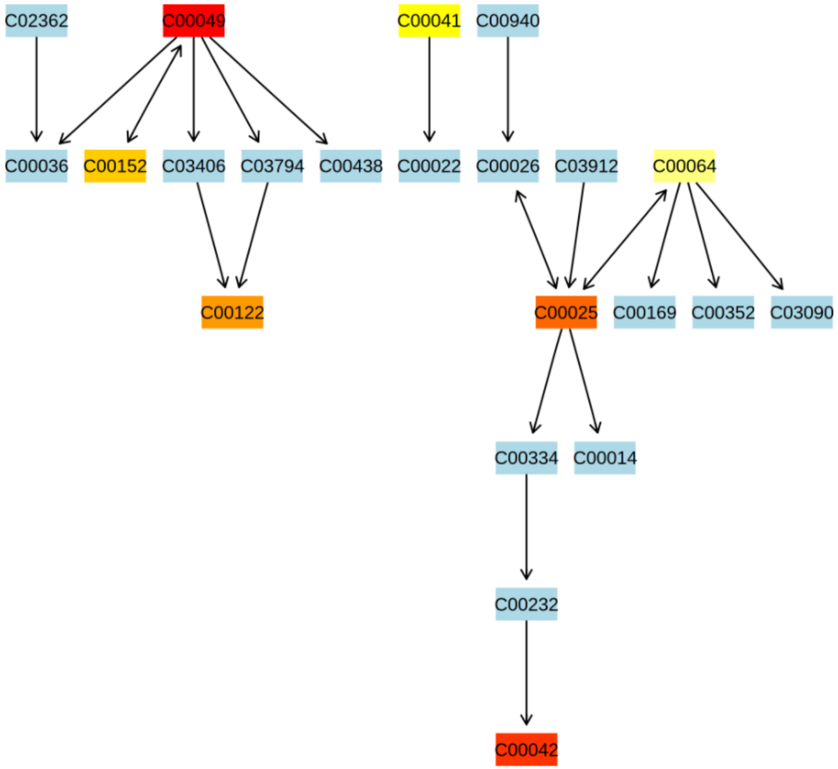

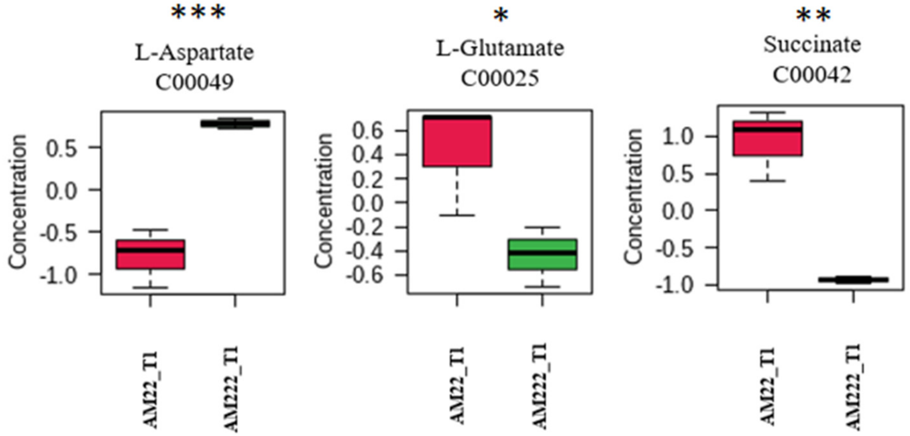

D. Shoot_T1_67-3_*vs*_AM22; Alanine, aspartate and glutamate metabolism FDR = 0.00020894 Impact = 0.64748

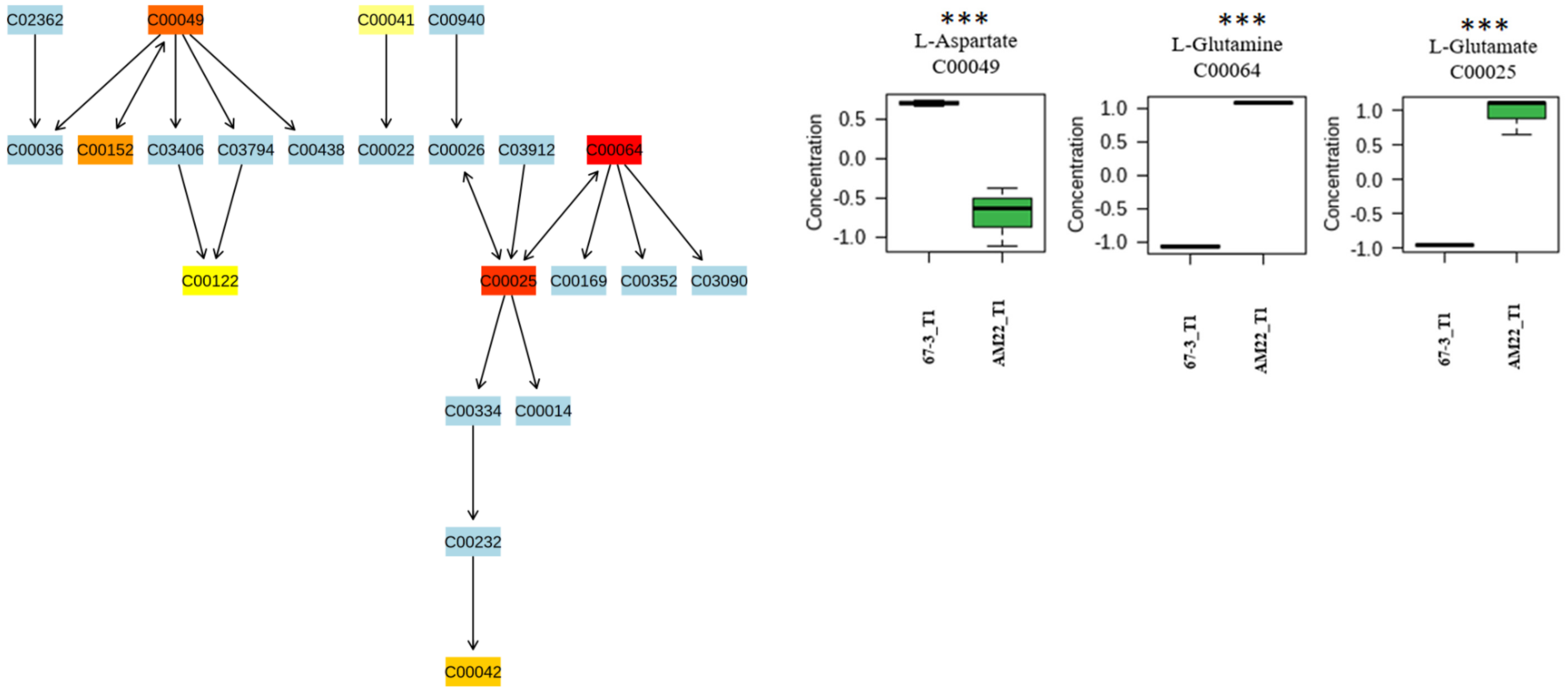

E. Shoot_T1_305E40_*vs*_AM22; Alanine, aspartate and glutamate metabolism FDR = 0.00029036 Impact = 0.64748

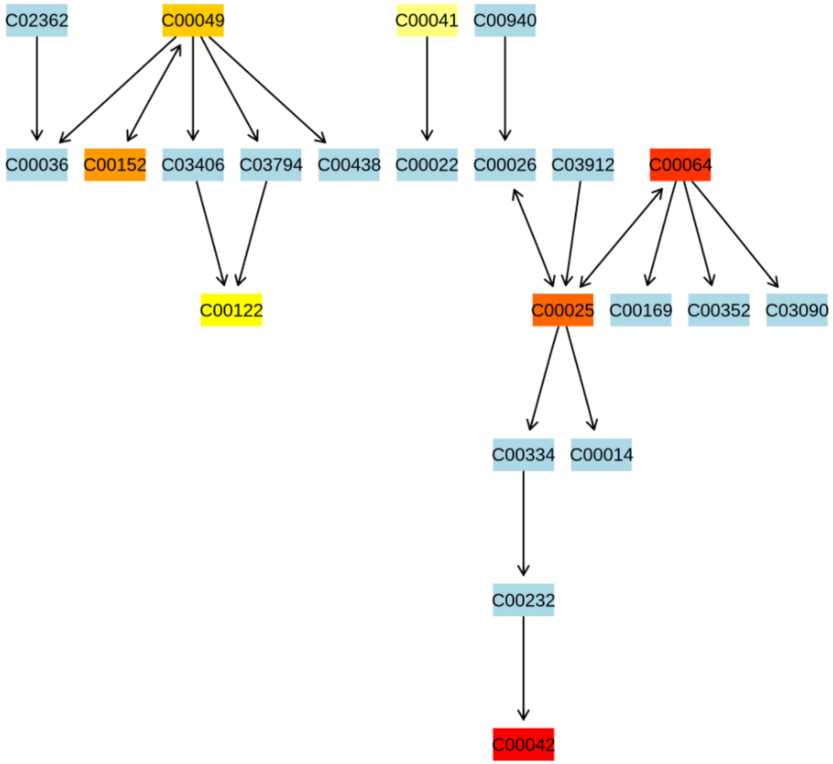

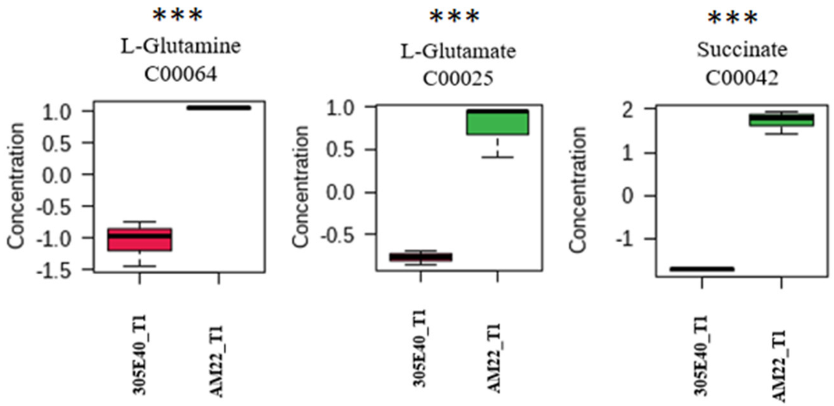

F. Shoot_T2_AM222_*vs*_AM22; Glyoxylate and dicarboxylate metabolism FDR = 0.018191 Impact = 0.28209

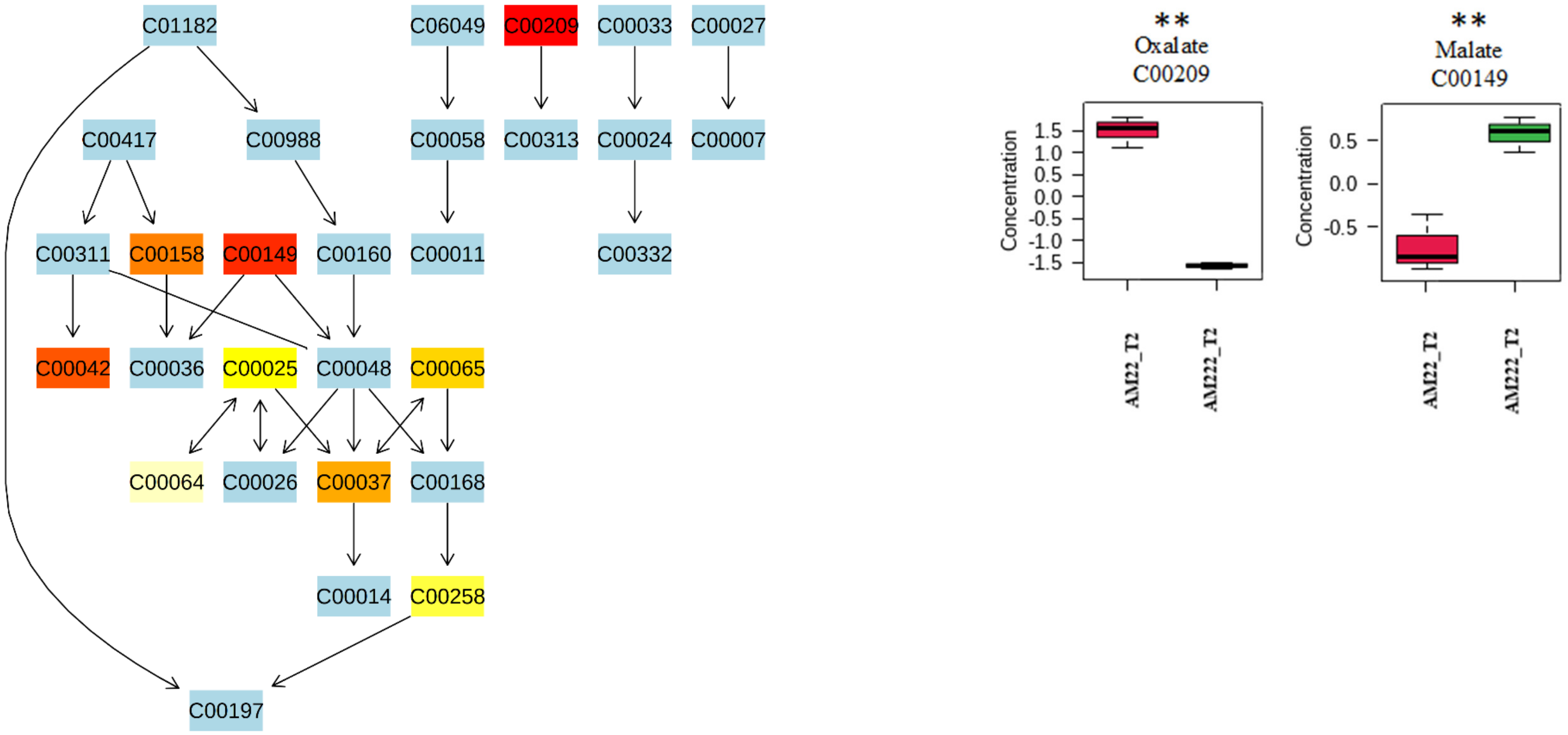

G. Shoot_T2_67-3_*vs*_AM22; Alanine, aspartate and glutamate metabolism FDR = 0.0032463 Impact = 0.64748

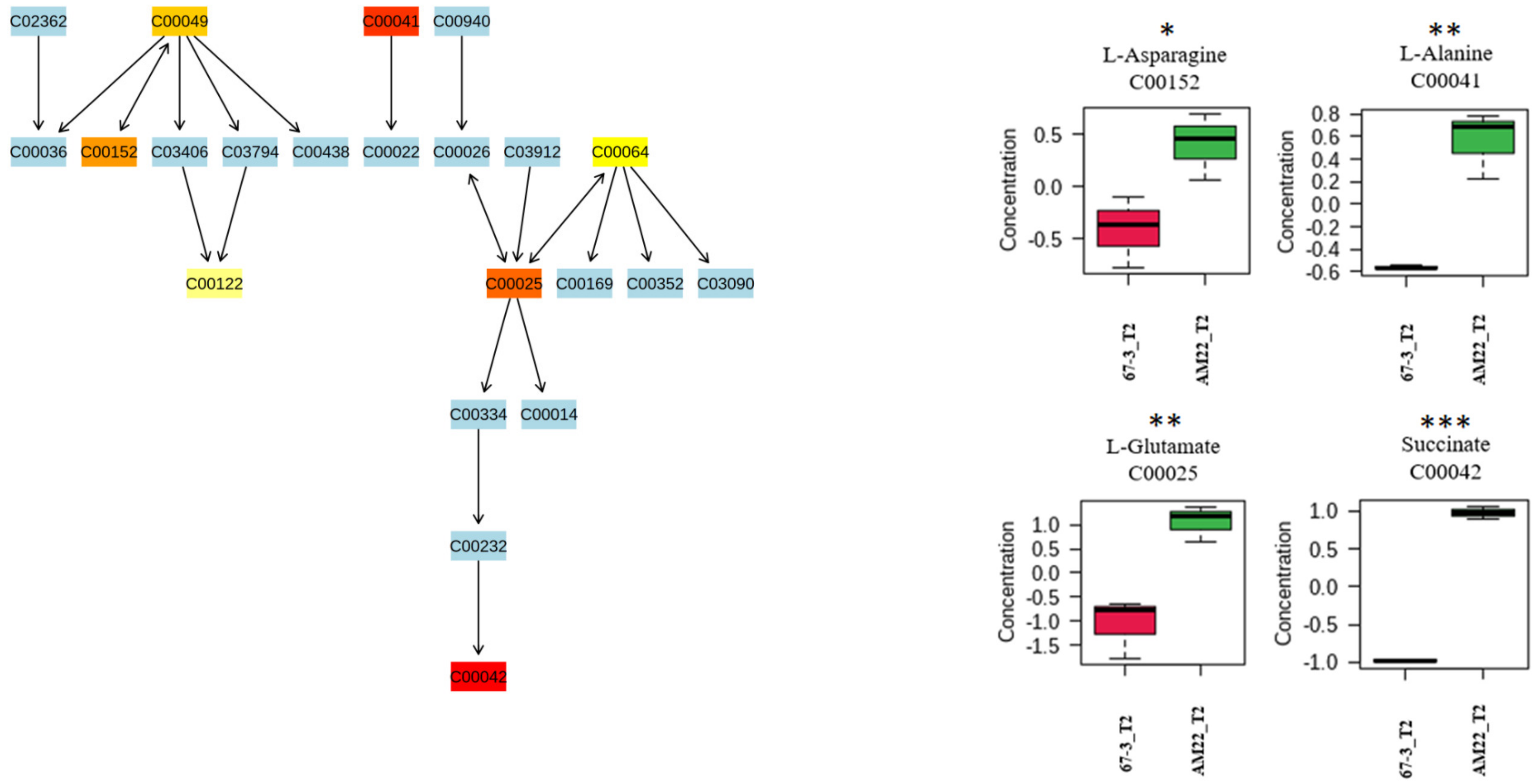

H. Shoot_T2_305E40_*vs*_AM22; Glycine, serine and threonine metabolism FDR = 0.0055903 Impact = 0.53598

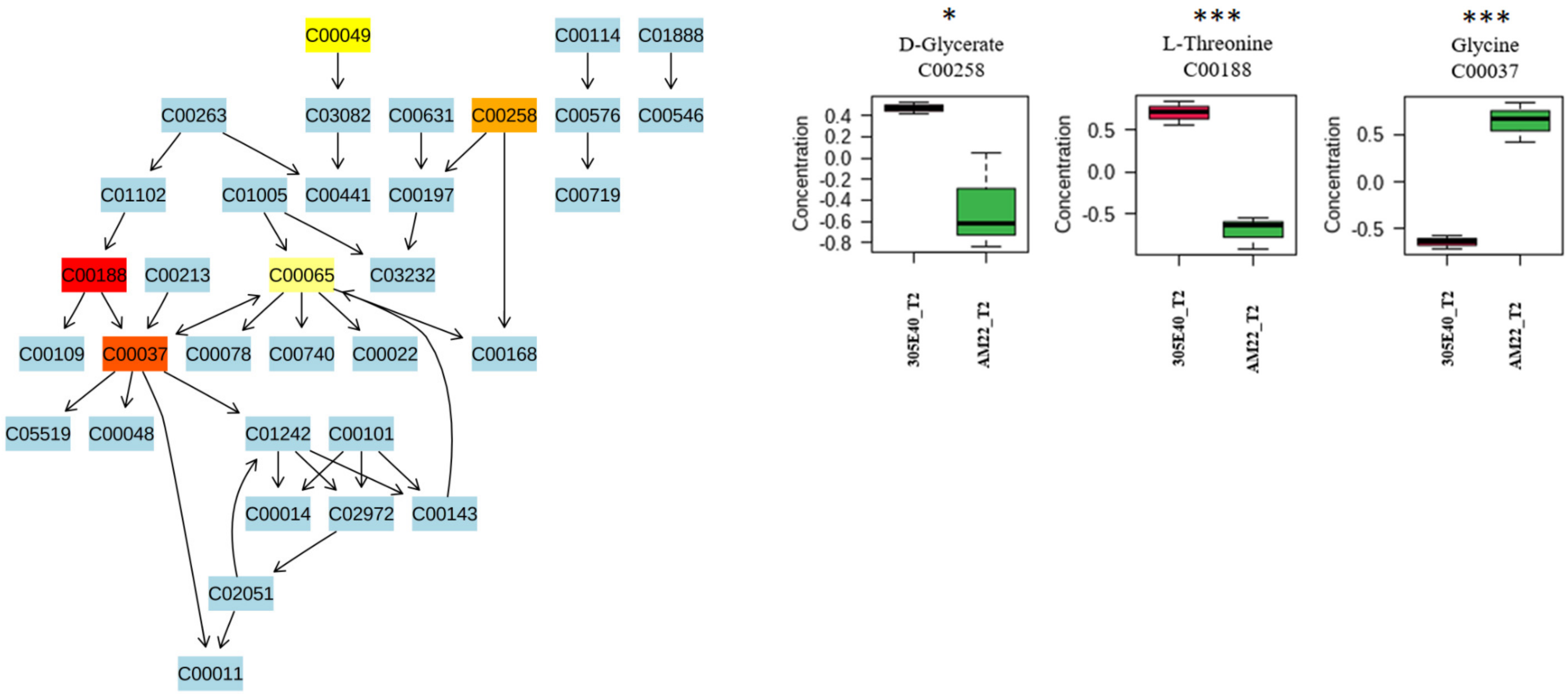

Figure S2. Correlation network between regulatory genes and related metabolites. The networks between metabolites and transcripts was performed with MetScape, bioinformatics framework for the visualization and interpretation of metabolomic data using Cytoscape software (version 3.8.1). Positive correlation is marked with red (A) and inverse correlation is marked with blue (B). Pathway enrichment analysis and pathway impact for both metabolites and metabolic genes (C).

A. Positive correlation

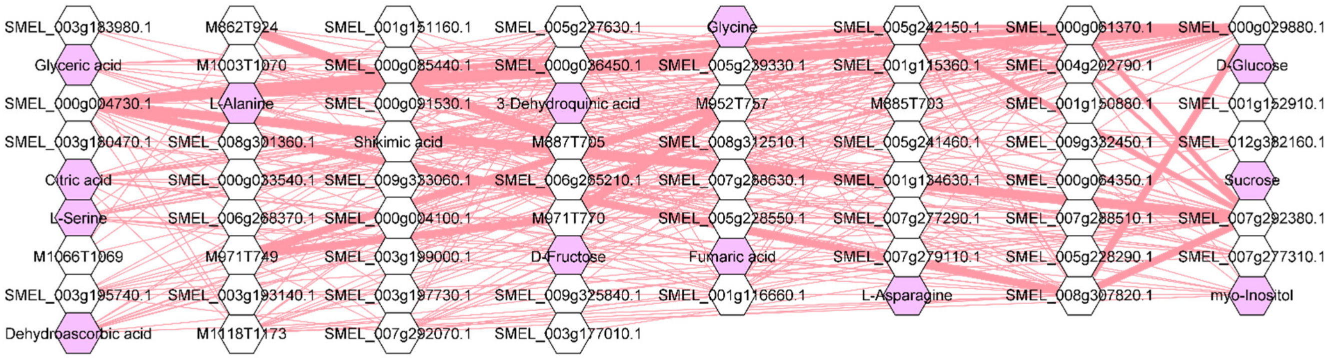

B. Negative correlation

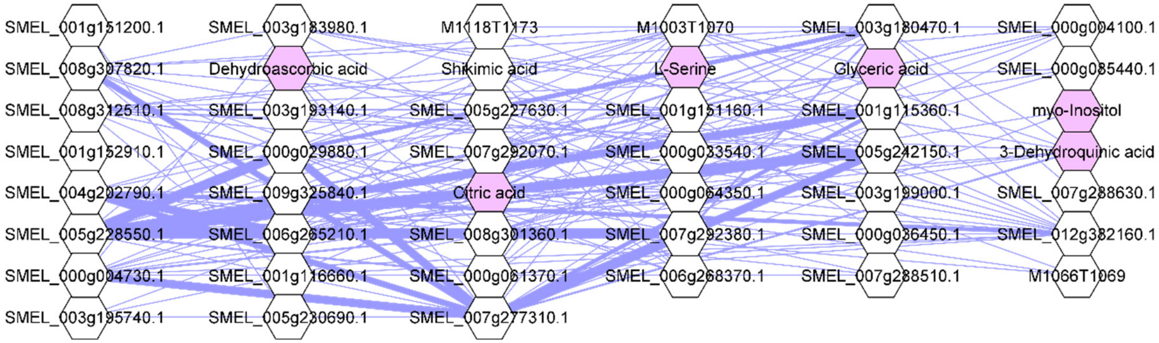

C. Pathway enrichment analysis and pathway impact

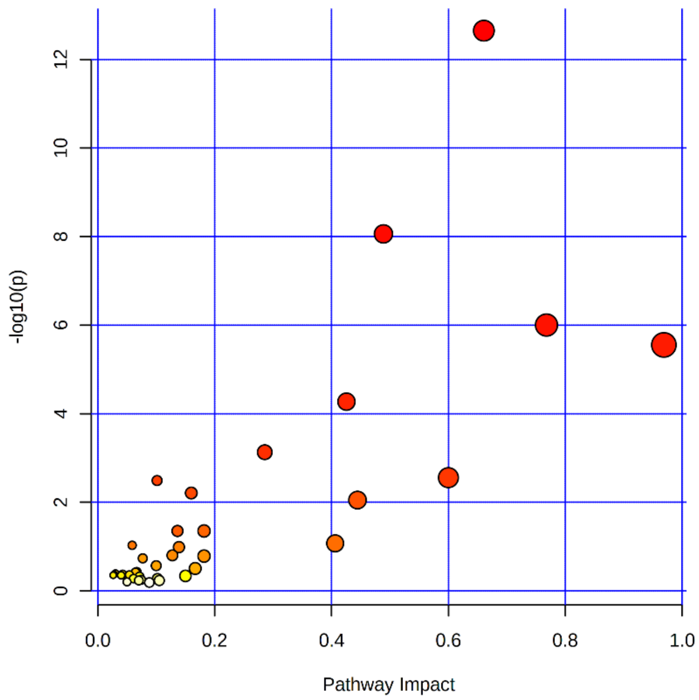

	Total	Expected	Hits	Raw p	-log(p)	Holm adjust	FDR	Impact
Glyoxylate and dicarboxylate metabolism	57	0.84534	13	2.24E-13	12.649	2.15E-11	2.15E-11	0.66071
Alanine, aspartate and glutamate metabolism	46	0.6822	9	8.70E-09	8.0605	8.27E-07	4.18E-07	0.48889
Starch and sucrose metabolism	57	0.84534	8	9.95E-07	6.0024	9.35E-05	3.18E-05	0.76786
Glycine, serine and threonine metabolism	65	0.96398	8	2.80E-06	5.5535	0.00026	6.71E-05	0.96875
Cyanoamino acid metabolism	48	0.71186	6	5.34E-05	4.2723	0.0049145	0.001026	0.42553
Butanoate metabolism	29	0.43008	4	0.000741	3.1304	0.067397	0.01185	0.28571
Citrate cycle (TCA cycle)	41	0.60805	4	0.002783	2.5556	0.25043	0.038161	0.6
Aminoacyl-tRNA biosynthesis	70	1.0381	5	0.00323	2.4909	0.28743	0.038754	0.10145

Figure S3. Heatmap of correlations in eggplant accession between metabolites and transcripts by the Pearson's correlation coefficient ($p < 0.05$) at T0. Correlations between eggplant metabolites and transcripts are based on 70 variables in shoot (A) and root (B).

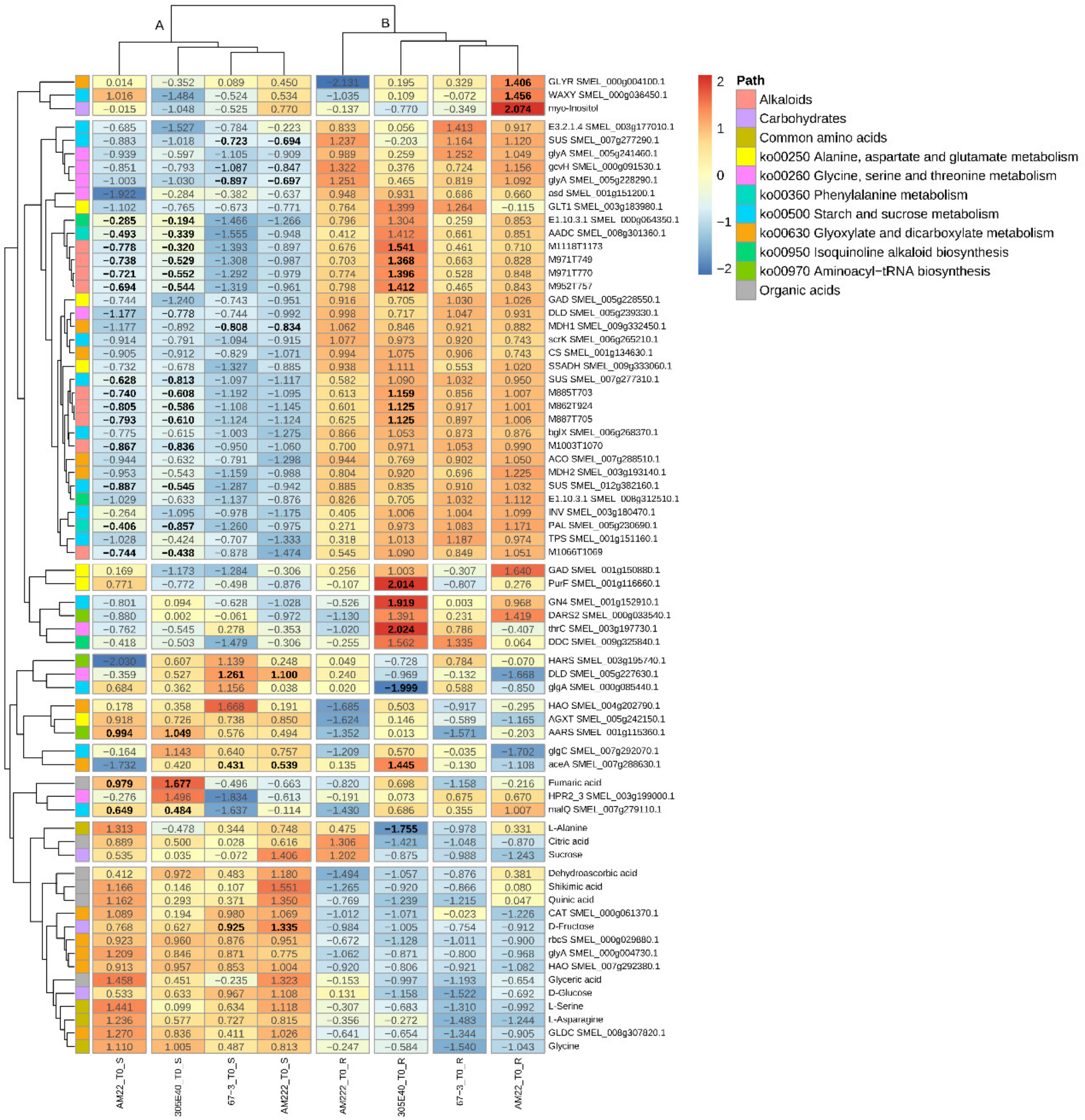

Figure S4. Heatmap of correlations in eggplant accession between metabolites and transcripts by the Pearson's correlation coefficient ($p < 0.05$) at T1. Correlations between eggplant metabolites and transcripts are based on 70 variables in shoot (A) and root (B).

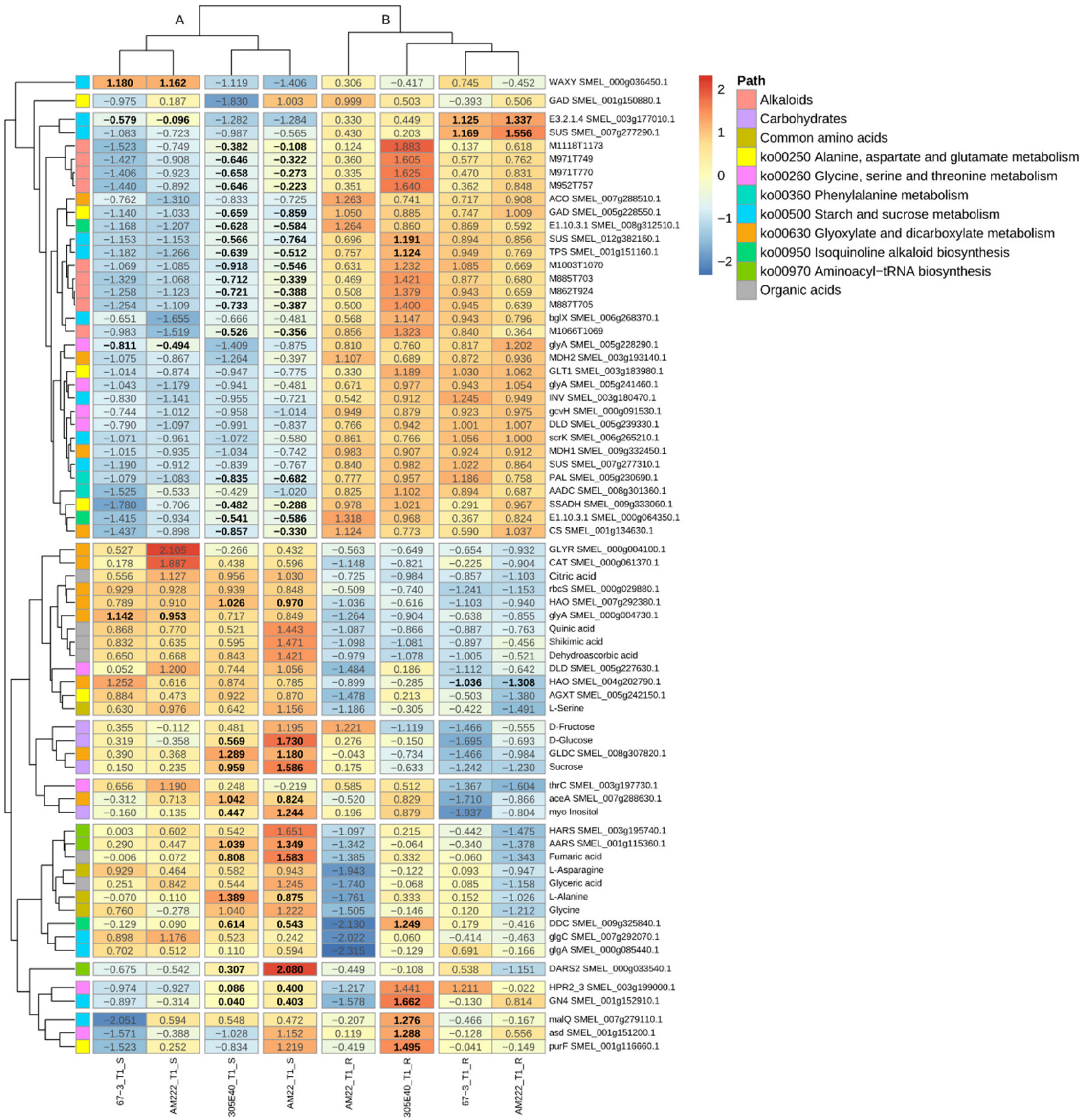

Figure S5. Heatmap of correlations in eggplant accession between metabolites and transcripts by the Pearson's correlation coefficient ($p < 0.05$) at T2. Correlations between eggplant metabolites and transcripts are based on 70 variables in shoot (A) and root (B).

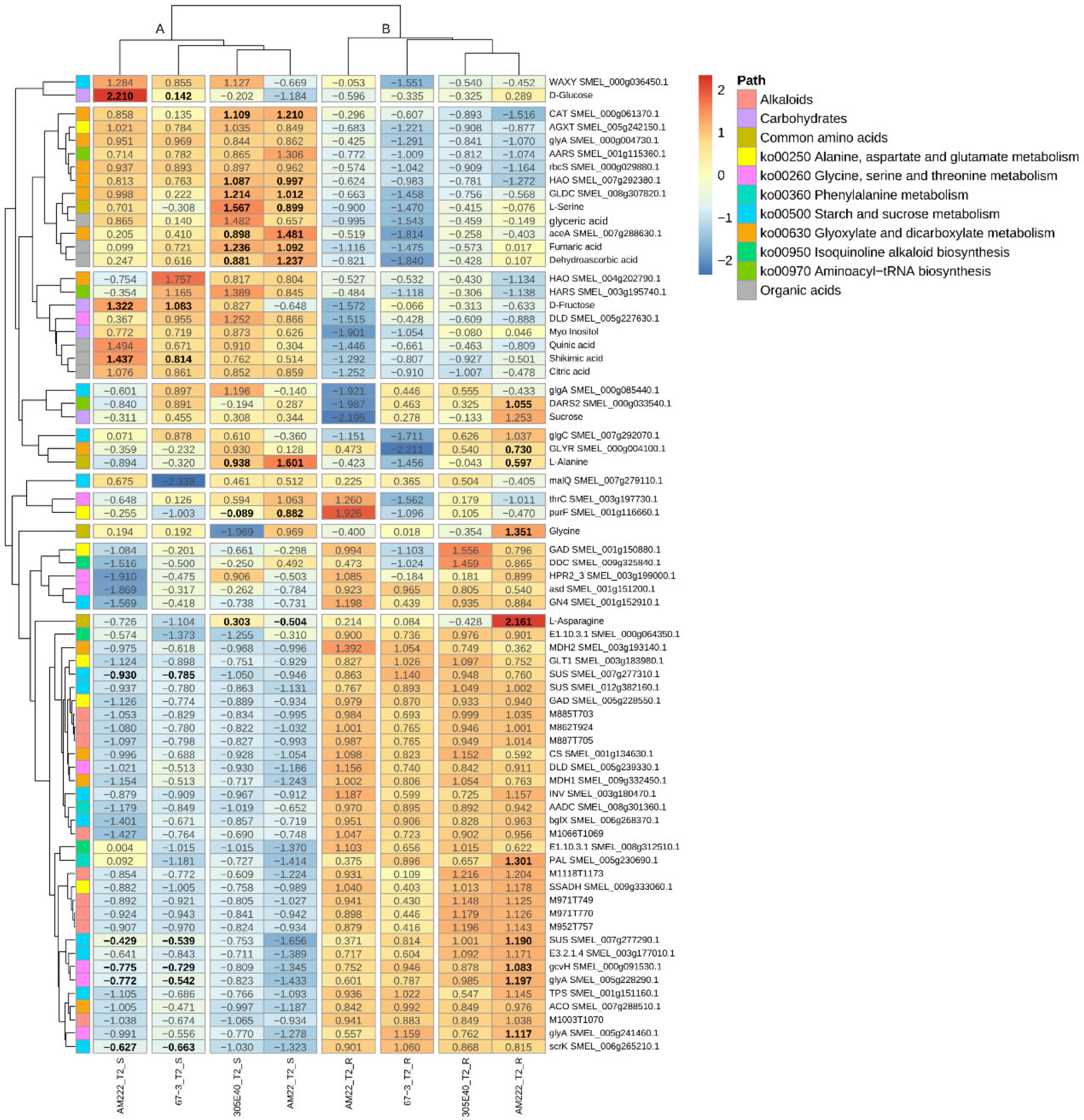

Figure S6. Exemplary scheme of Glycine, serine and threonine metabolism between efficient *vs* inefficient genotypes in shoots: A. T0, B. T1 and C. T2. In gray the metabolites and metabolic genes identified and not statistically different, in green the up-regulated and in yellow the down-regulated.

A. T0

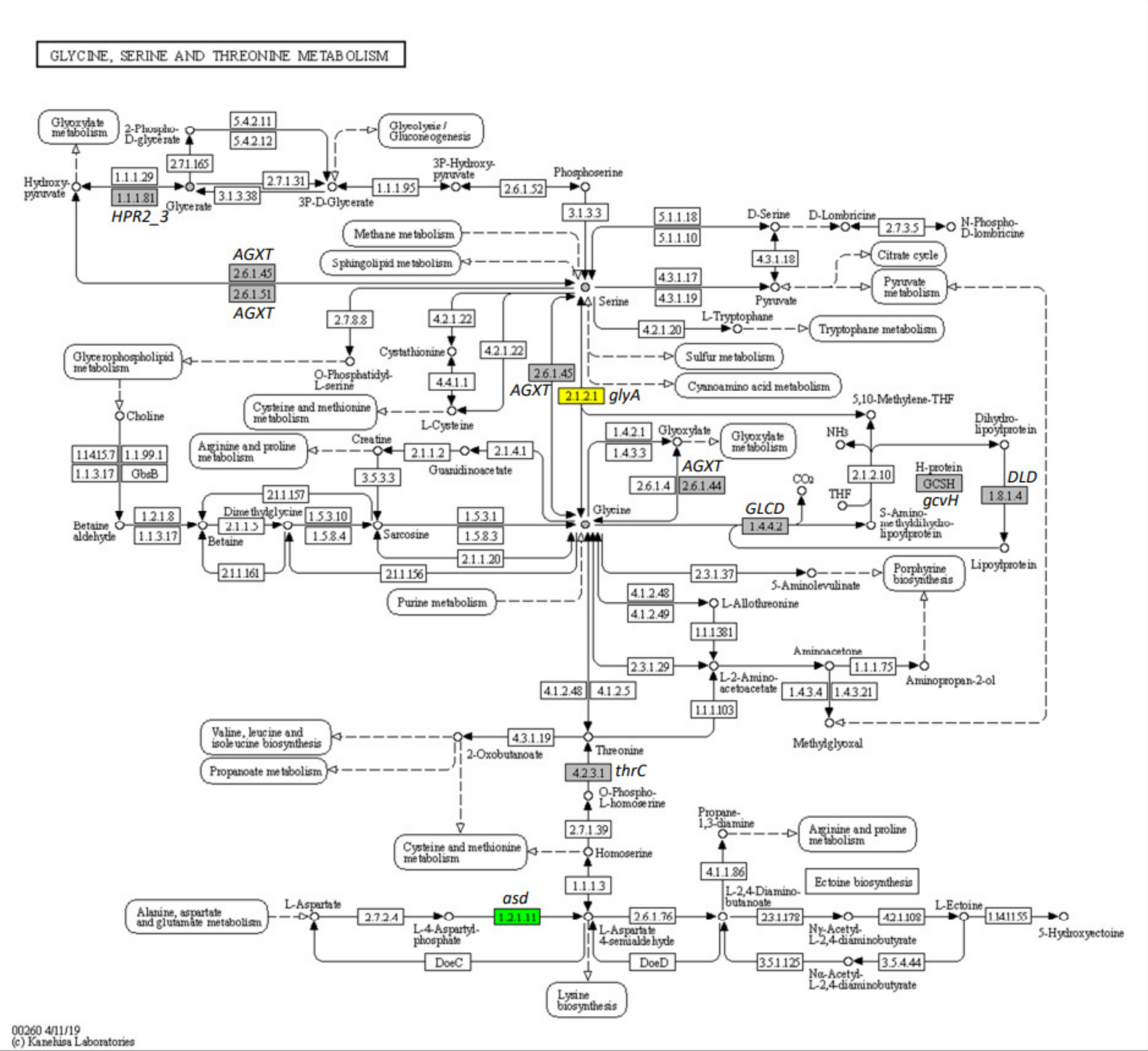

B. T1

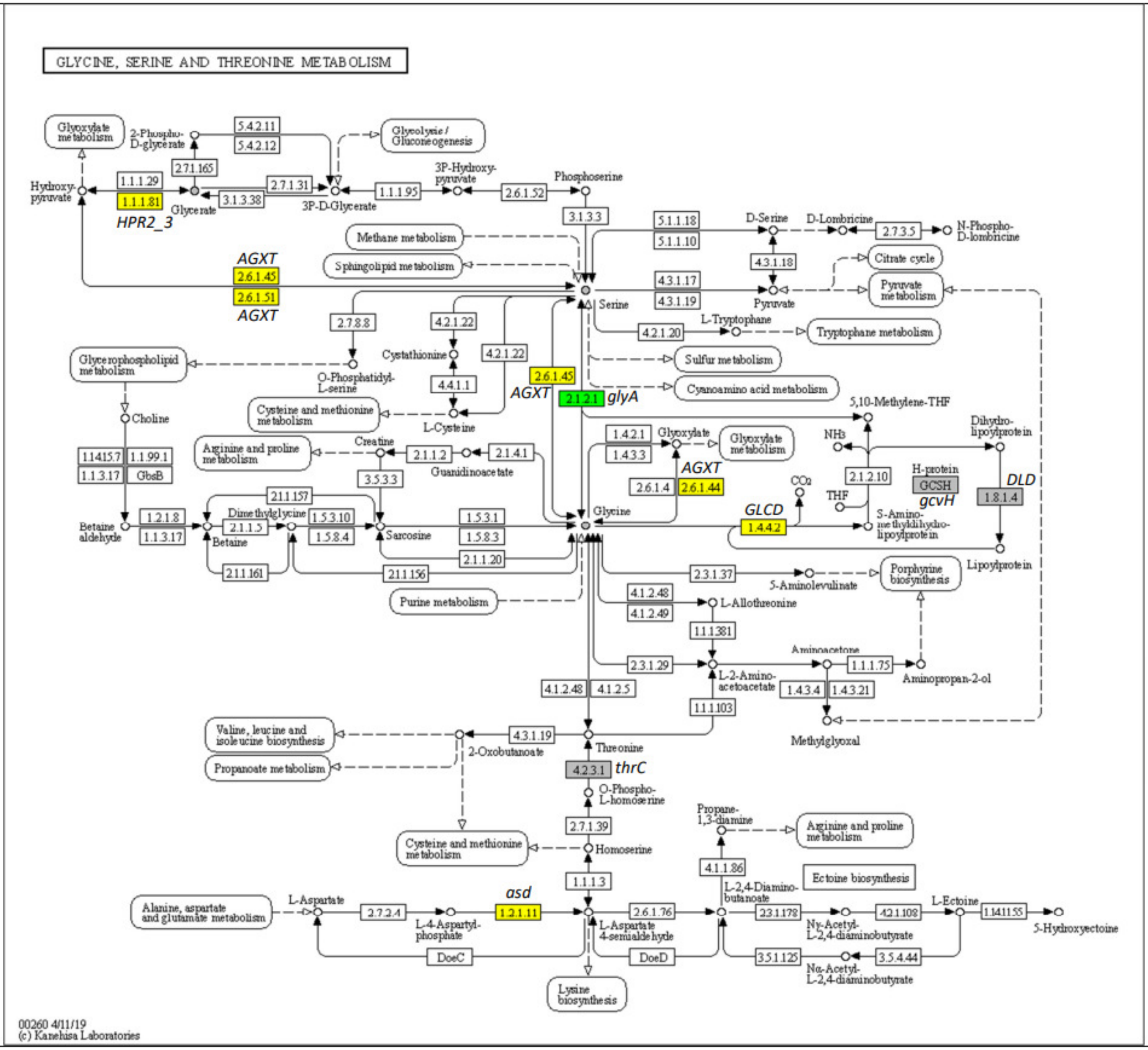
GLYCINE, SERINE AND THREONINE METABOLISM
HPR2_3
AGXT
glyA
GLCD
gcvH
DLD
thrC
asd
00260 4/11/19
(c) Kanehisa Laboratories

C. T2

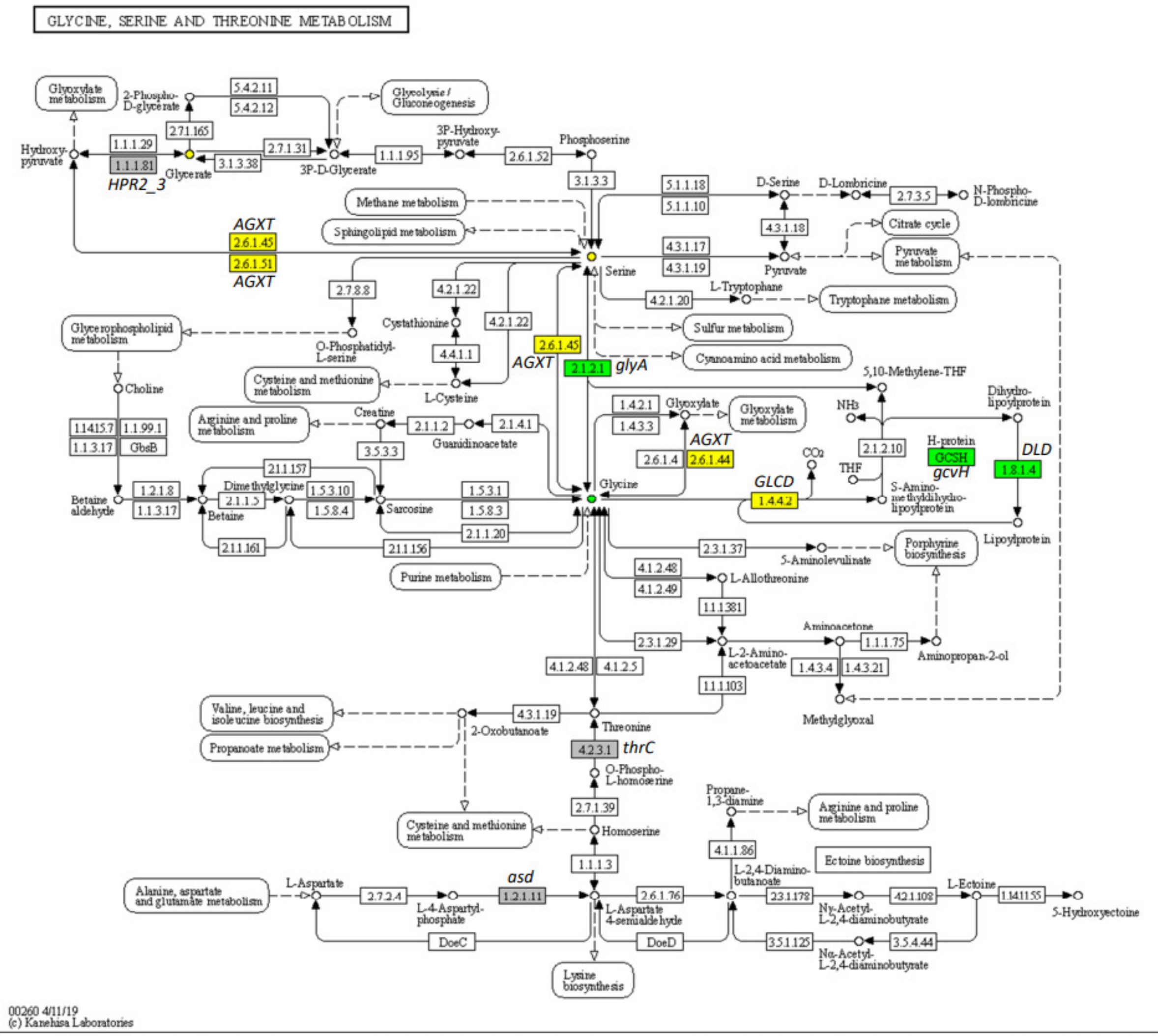
GLYCINE, SERINE AND THREONINE METABOLISM
Glyoxylate metabolism
2-Phospho-D-glycerate
5.4.2.11
5.4.2.12
Glycolysis / Gluconeogenesis
2.7.1.165
3P-Hydroxy-pyruvate
Phosphoserine
Hydroxy-pyruvate
1.1.1.29
1.1.1.81
2.7.1.31
3.1.3.38
1.1.1.95
2.6.1.52
Glycerate
3P-D-Glycerate
HPR2_3
3.1.3.3
5.1.1.18
5.1.1.10
D-Serine
D-Lombricine
2.7.3.5
N-Phospho-D-lombricine
Methane metabolism
Sphingolipid metabolism
4.3.1.18
Citrate cycle
AGXT
2.6.1.45
2.6.1.51
AGXT
4.3.1.17
4.3.1.19
Pyruvate
Pyruvate metabolism
Serine
2.7.8.8
4.2.1.22
L-Tryptophan
4.2.1.20
Tryptophan metabolism
Glycerophospholipid metabolism
Cystathionine
4.2.1.22
Sulfur metabolism
O-Phosphatidyl-L-serine
4.4.1.1
2.6.1.45
AGXT
2.1.2.1
glyA
Cyanoamino acid metabolism
5,10-Methylene-THF
Cysteine and methionine metabolism
L-Cysteine
Choline
Dihydro-lipoylprotein
1.4.2.1
1.4.3.3
Glyoxylate
Glyoxylate metabolism
NH3
Arginine and proline metabolism
Creatine
2.1.1.2
2.1.4.1
Guanidinoacetate
1.1.415.7
1.1.99.1
1.1.3.17
GbsB
3.5.3.3
AGXT
2.6.1.4
2.6.1.44
CO2
2.1.2.10
H-protein
GCSH
gcvH
DLD
1.8.1.4
THF
2.1.1.157
Dimethylglycine
1.5.3.10
GLCD
1.4.4.2
Glycine
S-Amino-methyldihydro-lipoylprotein
Betaine aldehyde
1.2.1.8
1.1.3.17
2.1.1.5
1.5.8.4
Betaine
Sarcosine
1.5.3.1
1.5.8.3
2.1.1.20
Lipoylprotein
2.1.1.161
2.1.1.156
2.3.1.37
5-Aminolevulinate
Porphyrine biosynthesis
Purine metabolism
4.1.2.48
4.1.2.49
L-Allothreonine
1.1.1.381
Aminoacetone
2.3.1.29
1.1.1.75
L-2-Amino-acetoacetate
Aminopropan-2-ol
4.1.2.48
4.1.2.5
1.4.3.4
1.4.3.21
1.1.1.103
Methylglyoxal
Valine, leucine and isoleucine biosynthesis
4.3.1.19
2-Oxobutanoate
Threonine
Propanoate metabolism
4.2.3.1
thrC
O-Phospho-L-homoserine
Propane-1,3-diamine
Arginine and proline metabolism
2.7.1.39
Cysteine and methionine metabolism
Homoserine
4.1.1.86
Ectoine biosynthesis
1.1.1.3
L-2,4-Diamino-butanoate
Alanine, aspartate and glutamate metabolism
L-Aspartate
asd
2.7.2.4
1.2.1.11
2.6.1.76
2.3.1.178
4.2.1.108
L-Ectoine
1.14.11.55
5-Hydroxyectoine
L-4-Aspartyl-phosphate
L-Aspartate 4-semialdehyde
Nγ-Acetyl-L-2,4-diaminobutyrate
DoeC
DoeD
3.5.1.125
3.5.4.44
Nα-Acetyl-L-2,4-diaminobutyrate
Lysine biosynthesis
00260 4/11/19
(c) Kanehisa Laboratories

Figure S7. Exemplary scheme of Glyoxylate and dicarboxylate metabolism between efficient vs inefficient genotypes in shoots: A. T0, B. T1, C. T2. In gray the metabolites and metabolic genes identified and not statistically different, in green the up-regulated and in yellow the down-regulated. In red the pathway modules relating to the Glyoxylate cycle and Photorespiration.

A. T0

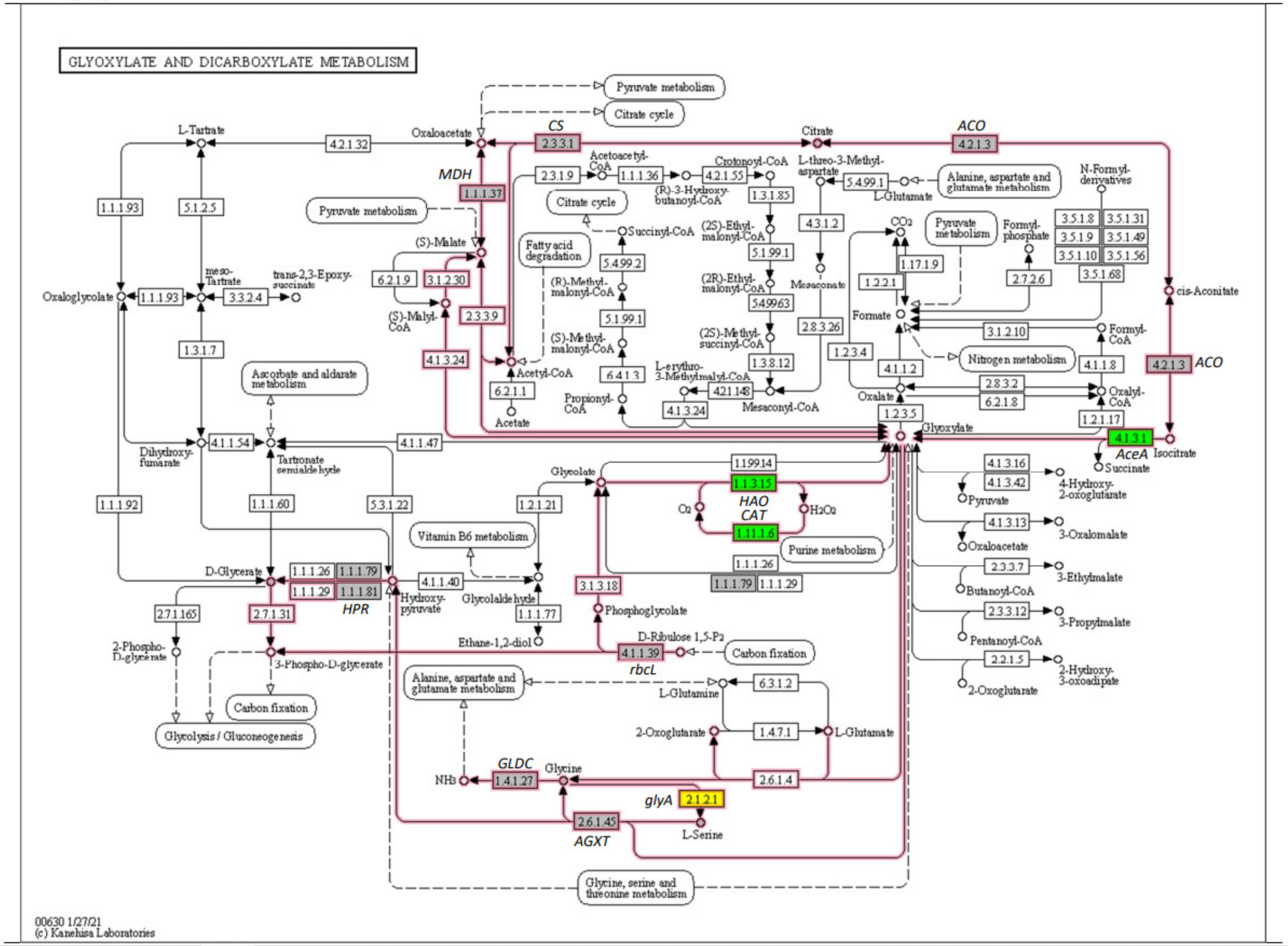

B. T1

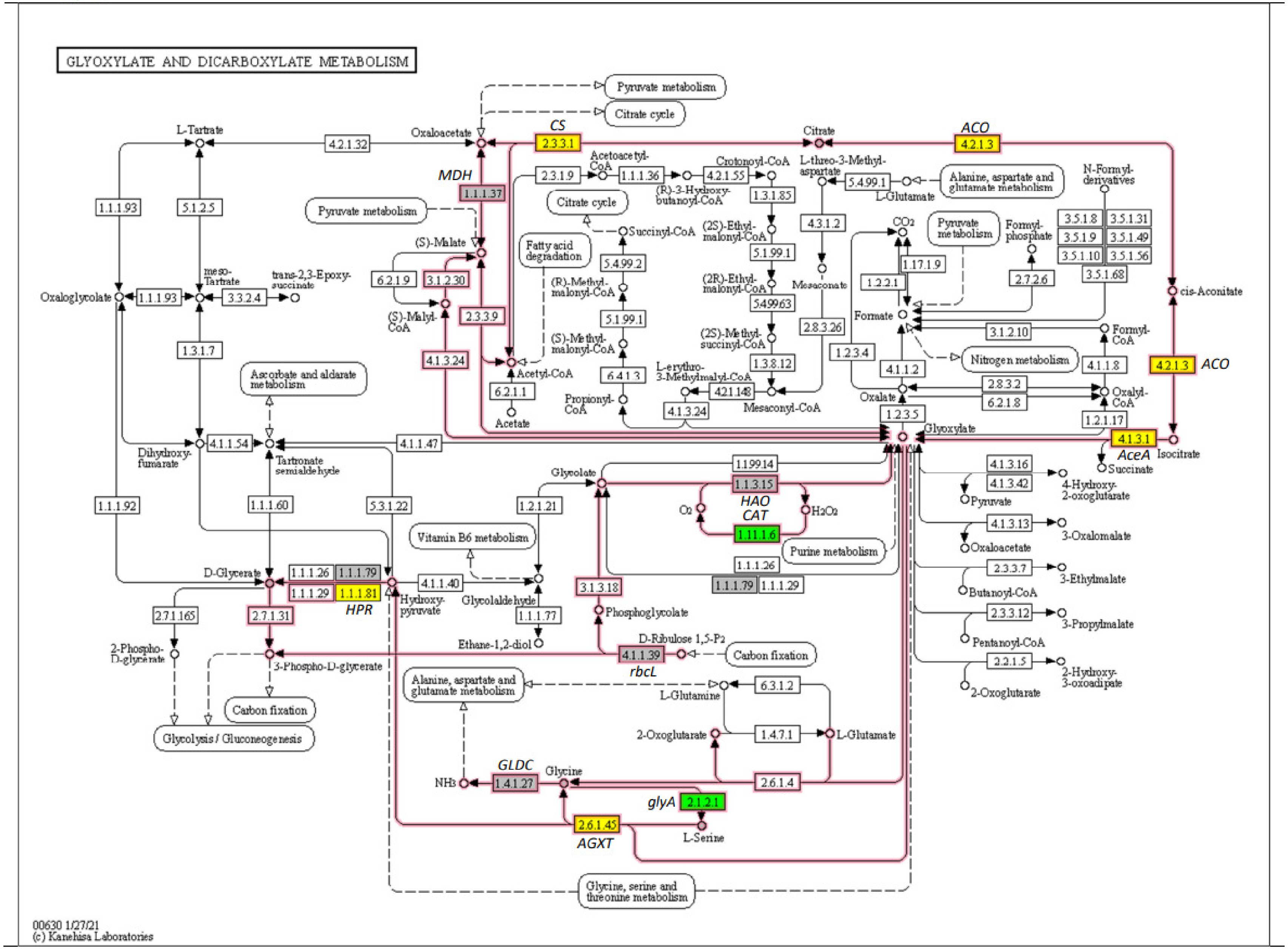

GLYOXYLATE AND DICARBOXYLATE METABOLISM
Pyruvate metabolism
Citrate cycle
L-Tartrate
4.2.1.32
Oxaloacetate
CS
2.3.3.1
Citrate
ACO
4.2.1.3
MDH
1.1.1.37
Acetoacetyl-CoA
2.3.1.9
1.1.1.36
(R)-3-Hydroxy-butanoyl-CoA
Crotonoyl-CoA
4.2.1.55
1.3.1.85
L-threo-3-Methyl-aspartate
5.4.99.1
L-Glutamate
Alanine, aspartate and glutamate metabolism
N-Formyl-derivatives
1.1.1.93
5.1.2.5
Pyruvate metabolism
Citrate cycle
(S)-Malate
Fatty acid degradation
Succinyl-CoA
(2S)-Ethyl-malonyl-CoA
4.3.1.2
CO2
1.17.1.9
Pyruvate metabolism
Formyl-phosphate
3.5.1.8
3.5.1.31
3.5.1.9
3.5.1.49
3.5.1.10
3.5.1.56
3.5.1.68
meso-Tartrate
trans-2,3-Epoxy-succinate
6.2.1.9
3.1.2.30
5.4.99.2
5.1.99.1
(R)-Methyl-malonyl-CoA
(2R)-Ethyl-malonyl-CoA
Mesaconate
1.2.1.2
2.7.2.6
Oxaloglycolate
1.1.1.93
3.3.2.4
(S)-Malyl-CoA
2.3.3.9
5.1.99.1
5.4.99.63
Formate
cis-Aconitate
2.8.3.26
3.1.2.10
Formyl-CoA
1.3.1.7
(S)-Methyl-malonyl-CoA
(2S)-Methyl-succinyl-CoA
4.1.3.24
Acetyl-CoA
1.2.3.4
Nitrogen metabolism
Ascorbate and aldarate metabolism
6.4.1.3
L-erythro-3-Methylmalyl-CoA
1.3.8.12
4.1.1.2
4.1.1.8
4.2.1.3
ACO
6.2.1.1
4.2.1.148
2.8.3.2
Propionyl-CoA
Oxalate
Oxalyl-CoA
6.2.1.8
Acetate
4.1.3.24
Mesaconyl-CoA
1.2.3.5
1.2.1.17
Glyoxylate
Dihydroxy-fumarate
4.1.1.54
4.1.1.47
4.1.3.1
AceA
Isocitrate
Tartronate semialdehyde
1.1.99.14
Succinate
Glycolate
1.1.3.15
4.1.3.16
HAO
4.1.3.42
4-Hydroxy-2-oxoglutarate
1.1.1.92
1.1.1.60
5.3.1.22
1.2.1.21
O2
CAT
H2O2
Pyruvate
1.11.1.6
4.1.3.13
Vitamin B6 metabolism
Purine metabolism
3-Oxalomalate
Oxaloacetate
1.1.1.26
D-Glycerate
1.1.1.26
1.1.1.79
2.3.3.7
1.1.1.79
1.1.1.29
3-Ethylmalate
4.1.1.40
Hydroxy-pyruvate
Glycolaldehyde
3.1.3.18
Butanoyl-CoA
1.1.1.29
1.1.1.81
HPR
2.7.1.165
2.7.1.31
1.1.1.77
Phosphoglycolate
2.3.3.12
3-Propylmalate
Pentanoyl-CoA
2-Phospho-D-glycerate
D-Ribulose 1,5-P2
Ethane-1,2-diol
Carbon fixation
4.1.1.39
3-Phospho-D-glycerate
rbcL
2.2.1.5
2-Hydroxy-3-oxoadipate
Alanine, aspartate and glutamate metabolism
6.3.1.2
L-Glutamine
2-Oxoglutarate
Carbon fixation
1.4.7.1
L-Glutamate
Glycolysis / Gluconeogenesis
GLDC
Glycine
NH3
1.4.1.27
2.6.1.4
glyA
2.1.2.1
2.6.1.45
AGXT
L-Serine
Glycine, serine and threonine metabolism
00630 1/27/21
(c) Kanehisa Laboratories

C. T2

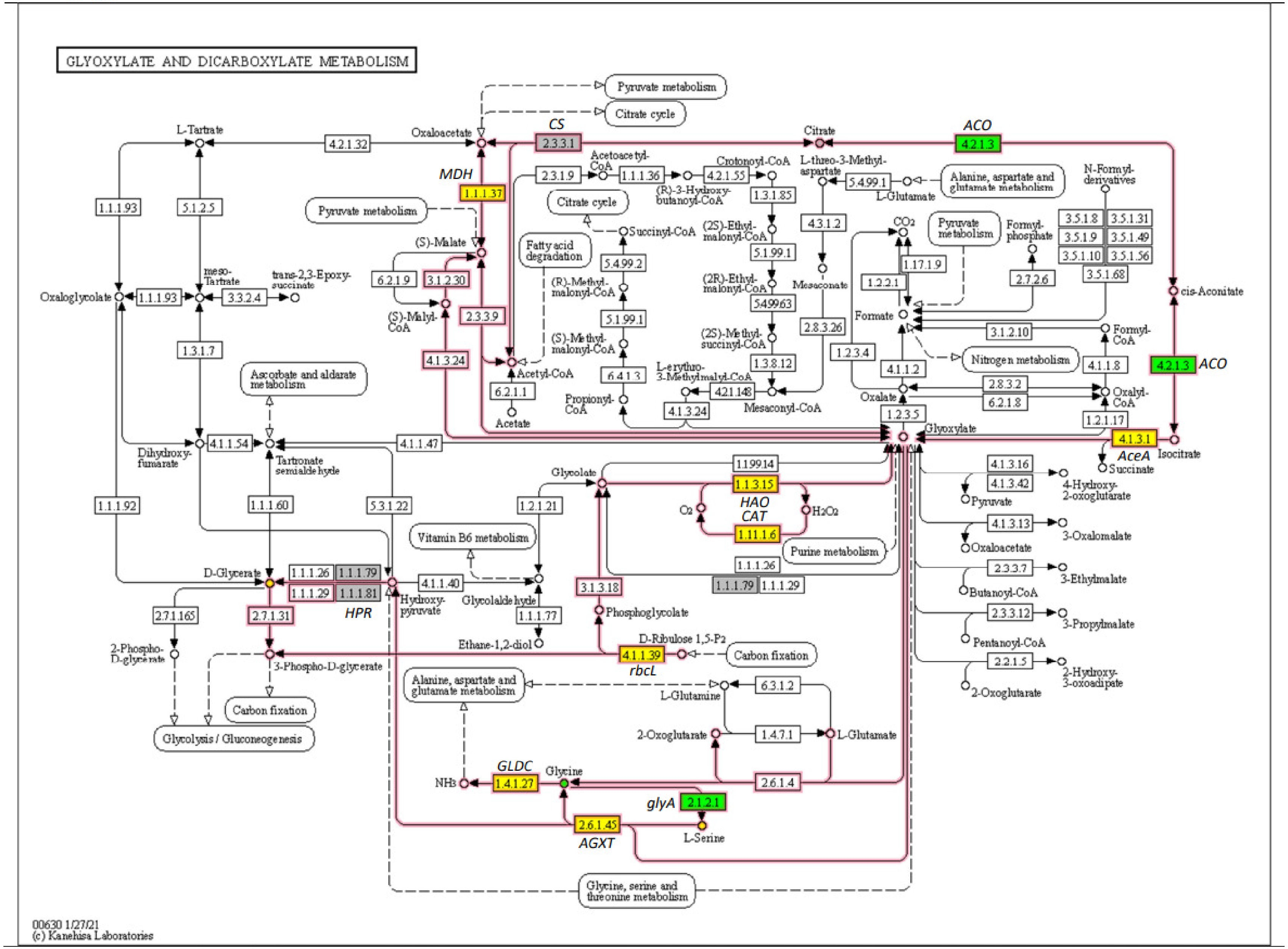
GLYOXYLATE AND DICARBOXYLATE METABOLISM
Pyruvate metabolism
Citrate cycle
L-Tartrate
4.2.1.32
Oxaloacetate
CS
2.3.3.1
Citrate
ACO
4.2.1.3
MDH
1.1.1.37
2.3.1.9
Acetoacetyl-CoA
1.1.1.36
(R)-3-Hydroxy-butanoyl-CoA
4.2.1.55
Crotonoyl-CoA
L-threo-3-Methyl-aspartate
5.4.99.1
L-Glutamate
Alanine, aspartate and glutamate metabolism
N-Formyl-derivatives
1.1.1.93
5.1.2.5
Pyruvate metabolism
Citrate cycle
1.3.1.85
Fatty acid degradation
Succinyl-CoA
(2S)-Ethyl-malonyl-CoA
4.3.1.2
CO2
Pyruvate metabolism
Formyl-phosphate
3.5.1.8
3.5.1.31
3.5.1.9
3.5.1.49
3.5.1.10
3.5.1.56
3.5.1.68
(S)-Malate
6.2.1.9
3.1.2.30
5.4.99.2
5.1.99.1
1.17.1.9
meso-Tartrate
trans-2,3-Epoxy-succinate
Oxaloglycolate
1.1.1.93
3.3.2.4
(S)-Malyl-CoA
2.3.3.9
(R)-Methyl-malonyl-CoA
(2R)-Ethyl-malonyl-CoA
Mesaconate
1.2.2.1
2.7.2.6
cis-Aconitate
5.4.99.63
5.1.99.1
Formate
3.1.2.10
Formyl-CoA
1.3.1.7
4.1.3.24
(S)-Methyl-malonyl-CoA
(2S)-Methyl-succinyl-CoA
2.8.3.26
Nitrogen metabolism
Ascorbate and aldarate metabolism
Acetyl-CoA
6.4.1.3
1.3.8.12
1.2.3.4
4.1.2
4.1.1.8
4.2.1.3
ACO
6.2.1.1
L-erythro-3-Methylmalyl-CoA
4.2.1.148
2.8.3.2
Oxalyl-CoA
Propionyl-CoA
Oxalate
6.2.1.8
Acetate
4.1.3.24
Mesaconyl-CoA
1.2.3.5
1.2.1.17
Dihydroxy-fumarate
4.1.1.54
4.1.1.47
Glyoxylate
4.1.3.1
Isocitrate
AceA
Succinate
Tartronate semialdehyde
1.1.99.14
Glycolate
4.1.3.16
4.1.3.42
4-Hydroxy-2-oxoglutarate
1.1.3.15
HAO
CAT
O2
H2O2
1.11.1.6
Pyruvate
1.1.1.92
1.1.1.60
5.3.1.22
1.2.1.21
4.1.3.13
3-Oxalomalate
Vitamin B6 metabolism
Purine metabolism
Oxaloacetate
D-Glycerate
1.1.1.26
1.1.1.79
1.1.1.29
1.1.1.81
4.1.1.40
1.1.1.26
2.3.3.7
3-Ethylmalate
1.1.1.79
1.1.1.29
Hydroxy-pyruvate
Glycolaldehyde
3.1.3.18
Butanoyl-CoA
2.7.1.165
2.7.1.31
HPR
1.1.1.77
Phosphoglycolate
2.3.3.12
3-Propylmalate
D-Ribulose 1,5-P2
Pentanoyl-CoA
2-Phospho-D-glycerate
Ethane-1,2-diol
4.1.1.39
Carbon fixation
3-Phospho-D-glycerate
rbcL
2.2.1.5
2-Hydroxy-3-oxoadipate
Alanine, aspartate and glutamate metabolism
6.3.1.2
L-Glutamine
2-Oxoglutarate
Carbon fixation
2-Oxoglutarate
1.4.7.1
L-Glutamate
Glycolysis / Gluconeogenesis
GLDC
Glycine
NH3
1.4.1.27
2.6.1.4
glyA
2.1.2.1
2.6.1.45
AGXT
L-Serine
Glycine, serine and threonine metabolism
00630 1/27/21
(c) Kanehisa Laboratories

Figure S8. Exemplary scheme of Starch and sucrose metabolism between efficient vs inefficient genotypes in shoots: A. T0, B. T1, C. T2. In gray the metabolites and metabolic genes identified and not statistically different, in green the up-regulated and in yellow the down-regulated. In red the pathway modules relating to the Glycogen biosynthesis and Trehalose biosynthesis.

A. T0

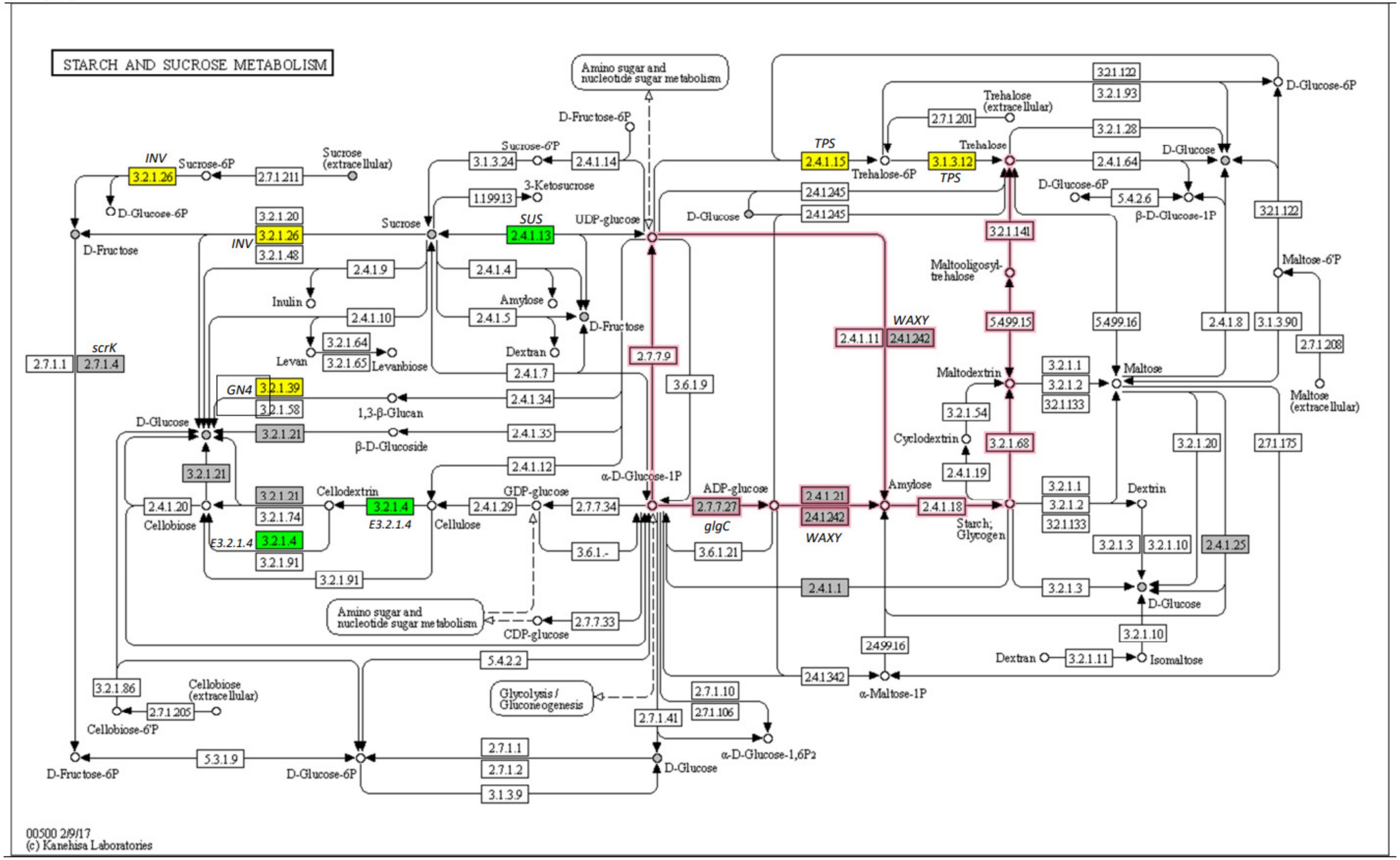

B. T1

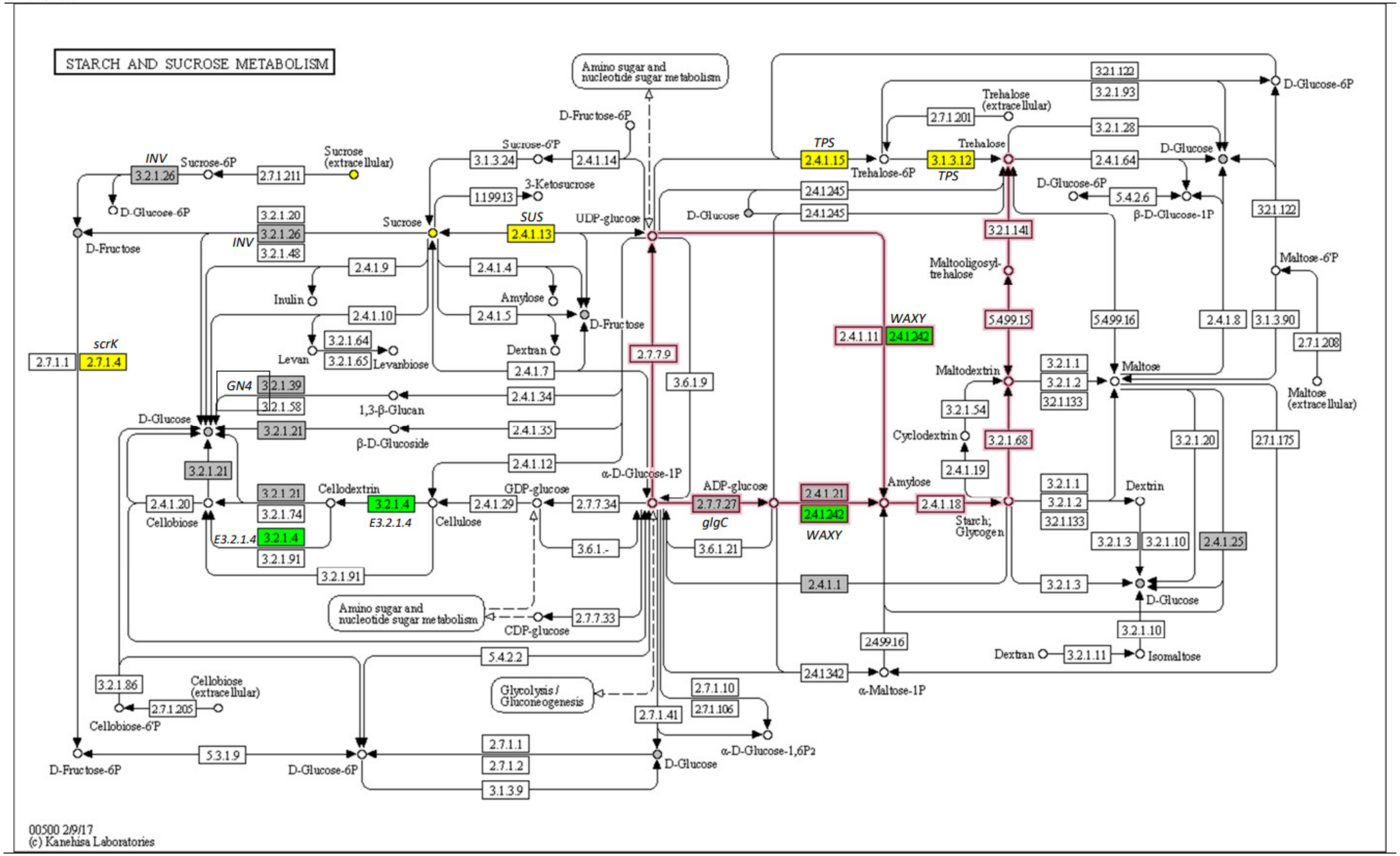
STARCH AND SUCROSE METABOLISM
Amino sugar and nucleotide sugar metabolism
D-Fructose-6P
Sucrose-6'P
3.1.3.24
2.4.1.14
3-Ketosucrose
1.1.99.13
INV
3.2.1.26
Sucrose-6P
2.7.1.211
Sucrose (extracellular)
D-Glucose-6P
3.2.1.20
3.2.1.26
3.2.1.48
INV
D-Fructose
Sucrose
SUS
2.4.1.13
UDP-glucose
2.4.1.9
2.4.1.4
Inulin
Amylose
2.4.1.10
2.4.1.5
D-Fructose
Levan
3.2.1.64
3.2.1.65
Levanbiose
Dextran
2.4.1.7
scrK
2.7.1.1
2.7.1.4
GN4
3.2.1.39
3.2.1.58
1,3-β-Glucan
2.4.1.34
D-Glucose
3.2.1.21
β-D-Glucoside
2.4.1.35
3.2.1.21
2.4.1.12
3.2.1.21
Cellodextrin
2.4.1.20
3.2.1.74
3.2.1.4
E3.2.1.4
Cellobiose
E3.2.1.4
3.2.1.4
3.2.1.91
3.2.1.91
Cellulose
2.4.1.29
GDP-glucose
2.7.7.34
3.6.1.-
2.7.7.33
CDP-glucose
Amino sugar and nucleotide sugar metabolism
5.4.2.2
3.2.1.86
Cellobiose (extracellular)
2.7.1.205
Cellobiose-6'P
D-Fructose-6P
5.3.1.9
D-Glucose-6P
2.7.1.1
2.7.1.2
3.1.3.9
D-Glucose
Glycolysis / Gluconeogenesis
2.7.1.41
2.7.1.10
2.7.1.106
α-D-Glucose-1,6P2
2.7.7.9
3.6.1.9
α-D-Glucose-1P
ADP-glucose
2.7.7.27
glgC
3.6.1.21
2.4.1.21
2.4.1.242
WAXY
2.4.1.1
2.4.1.342
α-Maltose-1P
2.4.99.16
Amylose
2.4.1.18
Starch; Glycogen
2.4.1.11
WAXY
2.4.1.242
TPS
2.4.1.15
Trehalose-6P
3.1.3.12
TPS
Trehalose
Trehalose (extracellular)
2.7.1.201
2.4.1.245
2.4.1.245
D-Glucose
3.2.1.141
Maltooligosyl-trehalose
5.4.99.15
Maltodextrin
3.2.1.54
Cyclodextrin
2.4.1.19
3.2.1.68
3.2.1.1
3.2.1.2
3.2.1.133
Maltose
5.4.99.16
3.2.1.1
3.2.1.2
3.2.1.133
Dextrin
3.2.1.3
3.2.1.10
2.4.1.25
3.2.1.3
D-Glucose
3.2.1.10
Dextran
3.2.1.11
Isomaltose
3.2.1.122
3.2.1.93
D-Glucose-6P
3.2.1.28
2.4.1.64
D-Glucose
D-Glucose-6P
5.4.2.6
β-D-Glucose-1P
3.2.1.122
Maltose-6'P
2.4.1.8
3.1.3.90
2.7.1.208
Maltose (extracellular)
3.2.1.20
2.7.1.175
00500 2/9/17
(c) Kanehisa Laboratories

C. T2

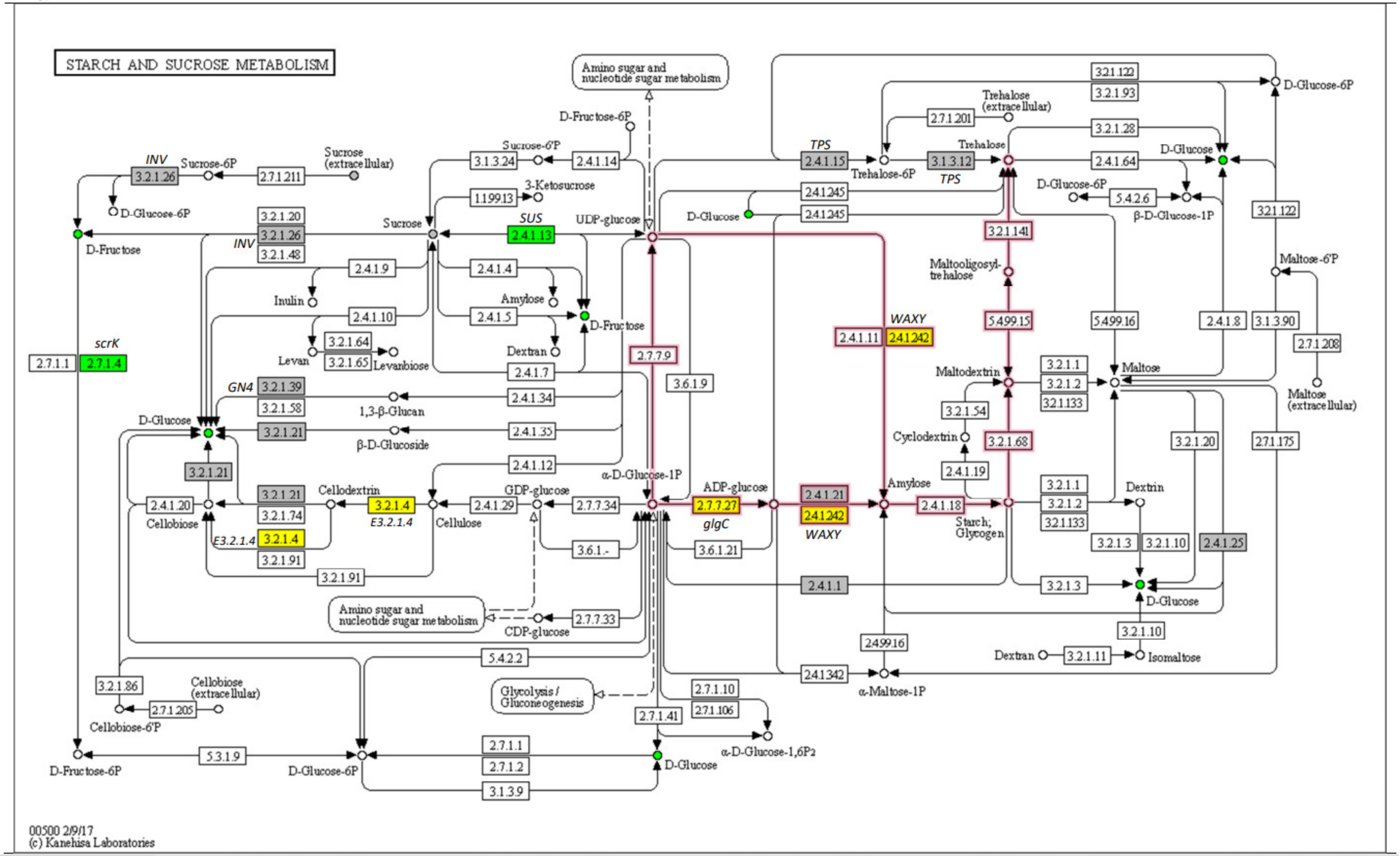
STARCH AND SUCROSE METABOLISM
Amino sugar and nucleotide sugar metabolism
D-Fructose-6P
Sucrose-6'P
3.1.3.24
2.4.1.14
INV
Sucrose-6P
3.2.1.26
2.7.1.211
Sucrose (extracellular)
D-Glucose-6P
1.1.99.13
3-Ketosucrose
3.2.1.20
INV
3.2.1.26
3.2.1.48
Sucrose
SUS
2.4.1.13
UDP-glucose
D-Fructose
2.4.1.9
2.4.1.4
Inulin
Amylose
2.4.1.10
2.4.1.5
D-Fructose
scrK
2.7.1.1
2.7.1.4
3.2.1.64
Levan
3.2.1.65
Levanbiose
Dextran
2.4.1.7
GN4
3.2.1.39
2.4.1.34
3.2.1.58
1,3-β-Glucan
D-Glucose
3.2.1.21
2.4.1.35
β-D-Glucoside
3.2.1.21
2.4.1.12
3.2.1.21
Cellodextrin
GDP-glucose
2.4.1.20
3.2.1.4
2.4.1.29
2.7.7.34
Cellobiose
E3.2.1.4
Cellulose
3.2.1.74
E3.2.1.4
3.2.1.4
3.6.1.-
3.2.1.91
3.2.1.91
Amino sugar and nucleotide sugar metabolism
2.7.7.33
CDP-glucose
5.4.2.2
3.2.1.86
Cellobiose (extracellular)
2.7.1.205
Cellobiose-6'P
Glycolysis / Gluconeogenesis
D-Fructose-6P
5.3.1.9
D-Glucose-6P
2.7.1.1
2.7.1.2
3.1.3.9
D-Glucose
2.7.1.41
2.7.1.10
2.7.1.106
α-D-Glucose-1,6P2
2.7.7.9
3.6.1.9
α-D-Glucose-1P
ADP-glucose
2.7.7.27
glgC
3.6.1.21
2.4.1.21
2.4.1.242
WAXY
2.4.1.1
2.4.1.342
2.4.99.16
α-Maltose-1P
Amylose
2.4.1.18
Starch; Glycogen
WAXY
2.4.1.11
2.4.1.242
TPS
2.4.1.15
Trehalose-6P
3.1.3.12
TPS
Trehalose
Trehalose (extracellular)
2.7.1.201
3.2.1.122
3.2.1.93
D-Glucose-6P
3.2.1.28
2.4.1.64
D-Glucose
D-Glucose
2.4.1.245
2.4.1.245
D-Glucose-6P
5.4.2.6
β-D-Glucose-1P
3.2.1.122
3.2.1.141
Maltooligosyl-trehalose
5.4.99.15
5.4.99.16
2.4.1.8
3.1.3.90
Maltose-6'P
2.7.1.208
Maltose (extracellular)
Maltodextrin
3.2.1.1
3.2.1.2
3.2.1.133
Maltose
3.2.1.54
Cyclodextrin
3.2.1.68
3.2.1.20
2.7.1.175
2.4.1.19
3.2.1.1
3.2.1.2
3.2.1.133
Dextrin
3.2.1.3
3.2.1.10
2.4.1.25
3.2.1.3
D-Glucose
3.2.1.10
Dextran
3.2.1.11
Isomaltose
00500 2/9/17
(c) Kanehisa Laboratories
